# Vascular biology of ageing—Implications in hypertension

**DOI:** 10.1016/j.yjmcc.2015.04.011

**Published:** 2015-06

**Authors:** Adam Harvey, Augusto C. Montezano, Rhian M. Touyz

**Affiliations:** Institute of Cardiovascular and Medical Sciences, BHF Glasgow Cardiovascular Research Centre, University of Glasgow, UK

**Keywords:** Vascular remodeling, Endothelial dysfunction, Oxidative stress, Mitochondria

## Abstract

Ageing is associated with functional, structural and mechanical changes in arteries that closely resemble the vascular alterations in hypertension. Characteristic features of large and small arteries that occur with ageing and during the development of hypertension include endothelial dysfunction, vascular remodelling, inflammation, calcification and increased stiffness. Arterial changes in young hypertensive patients mimic those in old normotensive individuals. Hypertension accelerates and augments age-related vascular remodelling and dysfunction, and ageing may impact on the severity of vascular damage in hypertension, indicating close interactions between biological ageing and blood pressure elevation. Molecular and cellular mechanisms underlying vascular alterations in ageing and hypertension are common and include aberrant signal transduction, oxidative stress and activation of pro-inflammatory and pro-fibrotic transcription factors. Strategies to suppress age-associated vascular changes could ameliorate vascular damage associated with hypertension. An overview on the vascular biology of ageing and hypertension is presented and novel molecular mechanisms contributing to these processes are discussed. The complex interaction between biological ageing and blood pressure elevation on the vasculature is highlighted. This article is part of a Special Issue entitled: CV Ageing.

## Highlights

• Ageing and hypertension are characterised by vascular dysfunction and remodelling.
• Molecular processes involve activation of MAP kinases and oxidative stress.
• Novel signalling molecules implicated in vascular ageing include FoxO and p66Shc.
• Biological ageing per se may not be fundamental in vascular changes of hypertension.

## Graphical abstract

Diagram demonstrating how vascular function and structure change with ageing and how this process impacts on development of hypertension. With increasing ageing and in the presence of compounding factors, such as environmental stressors, activation of the renin–angiotensin–aldsoterone system (RAAS) and/or activation of the sympathetic nervous system (SNS), molecular and cellular processes in the vasculature are impaired leading to endothelial dysfunction, vascular remodelling and inflammation. These events contribute to vascular injury, which leads to increased total peripheral resistance (TPR) in small arteries and increased stiffness in large arteries, contributing to increased blood pressure, which itself promotes vascular injury.

**Figure:**
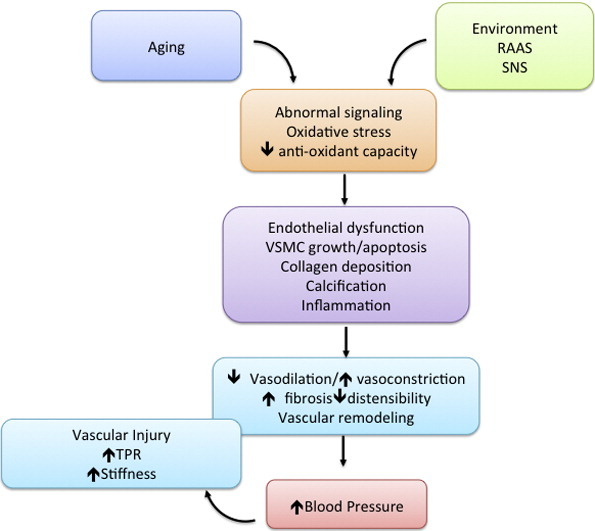

## 1 Introduction

**Fig. 1:**
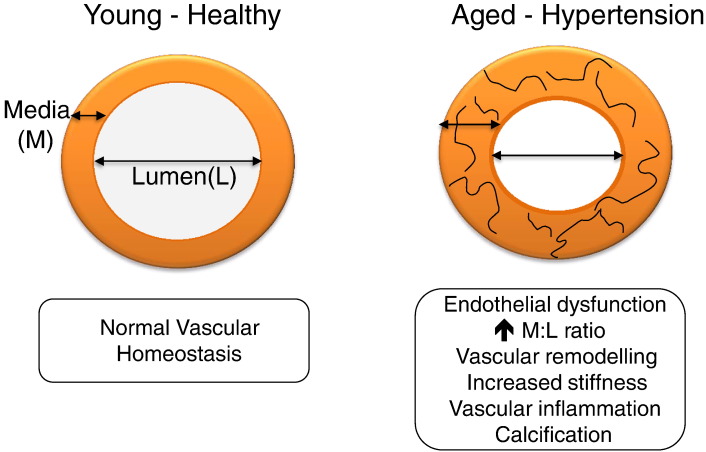
Schematic demonstrating vascular changes that occur during ageing and with the development of hypertension. Vascular changes in hypertension mimic those found in arteries observed with ageing.

Clinical studies show a significant relationship between ageing and increased blood pressure, with advancing age being a major non-modifiable risk factor in the development of hypertension [1]. This is due, in part, to changes that occur in the vasculature, including endothelial dysfunction, vascular remodelling, increased vascular stiffness and inflammation. These functional and structural changes define the ‘vascular phenotype’ of hypertension, features that are also found during ageing [2] (Fig. 1). At the cellular level, there is endothelial cell damage, increased vascular smooth muscle cell growth, cell migration, inflammation, contraction, extracellular matrix deposition, fibrosis, and calcification [3].

Young patients with elevated blood pressure exhibit arterial changes similar to those in older individuals with normal blood pressure, and accordingly the concept of ‘premature’ or ‘early’ vascular ageing in hypertension has been proposed [4]. Hypertension accelerates age-related vascular changes, processes that are attenuated when blood pressure is normalised. The direct relationship between ageing and vascular health is evident in progeria syndrome, where patients exhibit accelerated ageing, endothelial dysfunction, accelerated atherosclerosis and die prematurely from complications of cardiovascular disease, such as stroke and myocardial infarction [5]. Considering the fact that the population is ageing and that the major chronic disease of ageing is hypertension and associated cardiovascular complications, the potential health and economic burden in our modern society is enormous. Accordingly it is important to understand how vascular function changes with ageing and how this impacts on hypertension, so that targeted strategies could be developed to prevent and repair damaged ‘aged’ arteries and thereby reduce the risk of hypertension and target organ damage. In the present review, we discuss the vascular changes that occur with ageing and during the development of hypertension and focus on some molecular mechanisms that underlie these vascular changes.

## 2 Structural and mechanical changes in the ageing vasculature

Physiological changes to the vascular wall are dynamic and occur throughout life [6,7]. Endothelial cell turnover occurs over years, whereas that of vascular smooth muscle cells seems to occur over a shorter time period. Many structural and mechanical alterations have been observed in the aged vasculature including increased intimal-to-media (IM) thickness, evidenced by the finding that the IM thickness of the carotid artery increases two- to three-fold between 20 and 90 years of age [8,9]. Subclinical IM thickening is strongly associated with ageing and is also a predictor of future cardiovascular events [8,9]. Both aortic length and circumference gradually increase with advancing age [10–12]. Associated with these structural alterations are mechanical changes, characterised by a reduction in compliance, reduced elasticity/distensibility and increased stiffness [8,9]. Stiffening of the large conduit arteries due to fracture of elastin fibres within the tunica media and collagenous remodelling, results in increased aortic pulse pressure and pulse wave velocity (PWV). Increased PWV, a non-invasive measure of vascular stiffness, increases in both sexes with ageing and is determined by the mean arterial pressure and the intrinsic stress/strain relationship (stiffness) of the arterial wall. As arterial wall stiffness increases, central systolic pressure increases and diastolic pressure decreases, leading to increased pulse pressure, an independent risk factor for future cardiovascular events [13]. Processes underlying these structural and mechanical changes involve growth and migration of vascular smooth muscle cells within the media, vascular calcification and changes in the ratio of collagen and elastin in the vascular wall. Physiologically, the ratio of collagen and elastin remains constant due to their gradual production and degradation. In aged rodents the absolute elastin content of the aorta was shown not to differ to young counterparts. However, increased collagen content in 30-month-old animals compared to 6-month-old animals meant that the relative elastin content was decreased [14]. Collagen and elastin are regulated by catabolic matrix metalloproteinases (MMPs). Throughout ageing the balance between MMPs and their inhibitors (TIMPs) changes. For example, increased MMP-2 expression and activity in the vessels of old rats and non-human primates is increased compared to young counterparts [15,16].

## 3 Vascular calcification

Vascular calcification is a tightly controlled process similar to bone formation, where mineralization of the internal elastic lamina and elastic fibres in the media results in vascular stiffening. Calcification of the vascular media is a hallmark of vascular ageing [17]. Upregulation of transcription factors such as cbfa1 (core-binding factor 1α)/Runx2, MSX-2 and bone morphogenetic protein 2 (BMP-2), are involved in normal bone development and vascular calcification by regulating the expression of osteogenic proteins, including osteocalcin, osteonectin, alkaline phosphatase, collagen-1, and bone sialoprotein [18,19]. Another mechanism contributing to vascular mineralization is loss of calcification inhibitors, such as fetuin-A, matrix Gla protein, pyrophosphate, and osteopontin [19–21]. Molecular processes underlying this remain to be fully defined but increased expression of BMP2 and the osteoblast transcription factor Runx2/Cbfa1 [22], and modulation of Ca^2 +^ and Mg^2 +^ transport through cation channels, such as TRPM7 may represent important mediators in this process [23,24]. A correlation between age and vascular calcification has been described from 5% in individuals younger than 50 years to > 12% in individuals older than 80 years [25]. Ageing-associated vascular calcification has been reported in the aorta of rodents where the associated mechanisms include dysregulation of Matrix Gla protein [26]. Further possible mechanisms contributing to increased calcification with ageing includes dysregulation of vascular pyrophosphate [27,28]. Human studies have shown a weak inverse correlation between age and plasma pyrophosphate [29,30].

## 4 Ageing associated vascular inflammation

With ageing there is a shift towards a proinflammatory vascular phenotype with upregulation of inflammatory cytokines, chemokines and adhesion molecules in the vascular wall [8,9,31–38]. Pro-inflammatory transcription factors and proteins that have been identified in the ageing vascular media include MCP-1, TGF-β1, MMP-2, AP-1 and NF-kB [8,9,39]. Expression and activation of these molecules increases with ageing, processes that are usually associated with increased generation of reactive oxygen species (ROS). In aged arteries, there is downregulation of the transcription factor, nuclear factor (erythroid-derived 2)-like 2 (Nrf2), which stimulates expression of antioxidant enzymes, thereby leading to decreased anti-oxidant potential and increased ROS bioavailability with consequent oxidative stress [40]. Oxidative stress is a potent inducer of redox-sensitive pro-inflammatory signalling pathways, further contributing to inflammation and vascular damage with ageing [36–40].

## 5 Vascular contractility and ageing

Functionally, vascular contraction is altered during ageing and is determined in large part by changes in vascular smooth muscle cell cytoskeletal organisation and impaired contractile signalling. Mesenteric arteries from aged rats demonstrate hypercontractility in response to phenylephrine compared to young controls [40] an effect which is mirrored in the aorta [41]. These findings are paralleled in studies utilising the senescence-accelerated mouse (SAM-P8), which demonstrate increased vascular contractility in response to phenylephrine [42]. Conversely, studies performed on carotid vessels from aged guinea pigs displayed reduced contractile response to both phenylephrine and endothelin-1 (ET-1) compared to younger controls [43]. Thus it appears that differential responses during ageing may differ between species.

At the cellular level, with ageing, vascular smooth muscle cells, which are normally contractile, undergo phenotypic changes to become stiff and pro-migratory. Subsets of apoptotic, senescent and proliferative cells as well as hyper-contractile cells may co-exist in the vascular media. A major trigger for these functional changes is an increase in intracellular free Ca^2 +^ concentration ([Ca^2 +^]_i_), which occurs following activation of phopholipase C (PLC) leading to the generation of second messengers insitol trisphosphate (IP3) and diacylglycerol (DAG) [39,44]. Ca^2 +^ binds to calmodulin facilitating an interaction with myosin light chain kinase (MLCK) leading to its activation. Activated MLCK triggers phosphorylation of the regulatory light chains of myosin (MLC20) promoting cycling of myosin cross-bridges with actin and consequent contraction. Dephosphorylation of MLC20 by myosin light chain phosphatase (MLCP) results in VSMC relaxation. As such, the relative activities of MLCK and MLCP determine vascular smooth muscle tone by influencing the degree of MLC20 phosphorylation. Arteries from aged animals display altered responses to various contractile agents including norepinephrine, serotonin and KCL [44,45]. Mechanisms for this are incompletely understood, but the percentage of phosphorylated MLC20 induced by vasoactive agonists is different in young versus aged rats and may play a role in altered age-related contractile responses [45].

## 6 Endothelial function and ageing

The vascular endothelium is a monolayer of cells that lines blood vessels and plays a key role in arterial function through synthesis and release of biologically active molecules that can influence endothelial function in an autocrine or paracrine fashion. The healthy endothelium is characterised by a vasodilatory, anti-inflammatory and anti-thrombotic phenotype. Endothelial dysfunction is characterised by reduced vasodilatory responses to flow or agonists and is pro-inflammatory. Independent of the occurrence of other pathologies, ageing results in altered endothelium-dependent relaxation of both the aorta and resistance arteries in rodents [46,47]. These findings have been corroborated in human studies that suggest that endothelial function is gradually compromised with ageing [48,49]. A primary mechanism responsible for the deterioration of endothelial function with ageing is thought to be reduced bioavailability of the endothelium derived relaxing factor, nitric oxide (NO), due to its interaction with ROS to form peroxynitrite. Peroxynitrite oxidises BH4, an essential cofactor for NO synthesis by endothelial nitric oxide synthase (eNOS), to its inactive form resulting in reduced NO production. Furthermore, reduced BH4 can result in eNOS uncoupling whereby superoxide is produced in preference to NO. Reductions in BH4 levels have been reported in aged rodents [50].

## 7 Vascular signalling in ageing

**Fig. 2:**
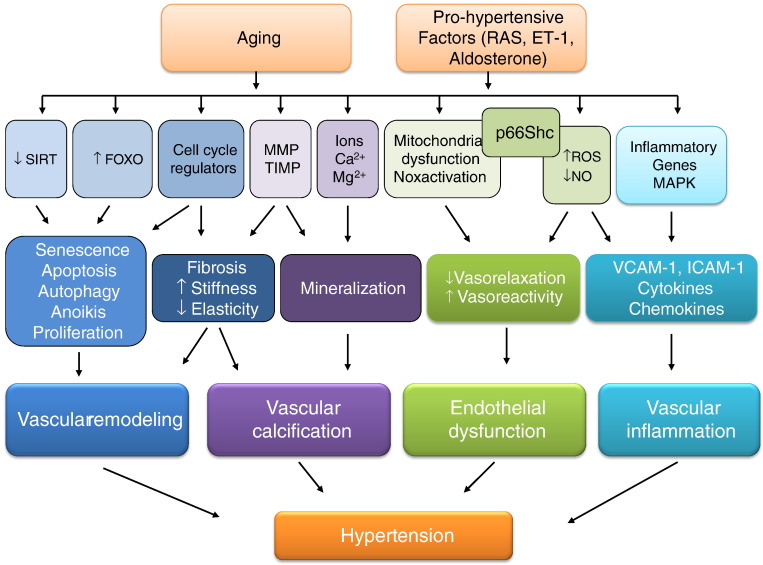
Molecular and cellular mechanisms associated with vascular changes in ageing and hypertension. Activation of pro-fibrotic, pro-inflammatory, redox-sensitive and growth/apoptotic signalling pathways lead to changes in vascular structure, mechanics and function with resultant arterial remodelling, calcification, inflammation, stiffness and impaired vasoreactivity. These vascular alterations are common features during ageing and in hypertension. VCAM-1, vascular cell adhesion molecule-1; ICAM-1, intercellular adhesion molecule-1; MMP, matrix metalloproteinases; TIMP, tissue inhibitor of metalloproteinase; RAS, renin angiotensin system; ET-1, endothelin-1; NO, nitric oxide.

Molecular mechanisms and cell signalling events underlying the structural and functional alterations observed during ageing are similar to those that occur in hypertension (Fig. 2). Many age/longevity-related molecules and signalling cascades have been described, of which a few of the novel systems are highlighted below.

### 7.1 Sirtuins

Sirtuins (SIRTs) are a family of NAD-dependent protein deacetylases and ribosyl transferases consisting of 7 members which are localised in the cytoplasm (SIRT1 and SIRT2), nucleus (SIRT1, SIRT2, SIRT 6 and SIRT 7) or mitochondria (SIRT3, SIRT4 and SIRT 5). SIRTs have been implicated in various cellular processes associated with ageing, including, apoptosis, inflammation and mitochondrial biogenesis. SIRTs are able to modulate the ageing process in a number of species [51–53]. This is highlighted by the following: 1) SIRT1 protects against phosphate-induced arterial calcification, possibly due to the inhibition of osteoblastic transdifferentiation [54]; 2) mitochondrial localised SIRT3 regulates many proteins that are important in the regulation of mitochondrial function including pyruvate dehydrogenase, SOD2 and cyclophilin D; 3) SIRT3 −/− mice exhibit accelerated cardiovascular ageing [55] and 4) SIRT3 has vasoprotective effects through interaction with FOXO3, which enhances mitochondrial antioxidant defence systems [56].

### 7.2 PGC-1α

Another emerging candidate implicated in age-related signalling in the vasculature, is peroxisome proliferator-activated receptor gamma coactivator-1α (PGC-1α), which plays an important role in regulating mitochondrial biogenesis and turnover [57]. Because mitochondria require continuous recycling and regeneration throughout the lifespan and are subject to continuous damage over time, regulation of mitochondrial biogenesis and turnover is critical for maintained energy production and prevention of oxidative damage, and the promotion of healthy ageing. Impaired mitochondrial biogenesis is an important inducer of age-related changes in the endothelium and vascular smooth muscle [58–60]. The aged vasculature displays reduced levels of PGC-1α with consequent mitochondrial dysregulation of the electron transport chain and other mitochondrial proteins leading to oxidative stress and vascular injury [60]. Decreased AMPK activity may contribute to reduced PGC-1α activation and impaired mitochondrial function associated with ageing [61].

### 7.3 FoxO transcription factors

The FoxO family of Forkhead transcription factors are involved in tumour suppression, energy metabolism, and longevity. Mammals express four FoxO isoforms, FoxO1, FoxO3, FoxO4 and FoxO6. FoxO1, FoxO3 and FoxO4 are phosphorylated in an Akt-dependent manner that promotes FoxO export from the nucleus to the cytoplasm, thereby repressing FoxO transcriptional function. FoxO targets include genes that have pivotal roles in cell cycle progression (p21, p27) and ROS detoxification (MnSOD) and thus may be important in regulation of the ageing phenotype in the vasculature [62,63]. FoxO3 is a direct target of SIRT3 deacetylation protecting mitochondria against age-related oxidative stress and promoting upregulation of genes that are essential for mitochondrial homeostasis [64]. Several reports have suggested that FoxO3 may be a determinant of ageing, due to the fact that single-nucleotide polymorphisms in the FoxO3 gene are associated with longevity in humans [65,66]. FoxO3 knockout mice however do not exhibit reduced lifespan [67], and as such, the exact role of FoxO3 in longevity and ageing still remains unclear.

### 7.4 p66shc

Mitochondrial dysfunction and increased mitochondrial-derived ROS have been implicated in vascular changes in ageing [68]. An important mediator of mitochondrial ROS production and thus regulator of the intracellular pathways that govern oxidative stress, apoptosis, and cell growth/survival is the adapter protein p66shc. p66Shc is phosphorylated at serine 36 by PKC*β* and VEGF, resulting in recognition by the prolyl isomerase Pin1, allowing translocation and entrance into mitochondria where it interacts with cytochrome C resulting in production of H_2_O_2_. Levels of p66shc in heart, kidney and vascular smooth muscle increase with ageing [69]. Mice lacking p66shc gene display a 30% increase in lifespan compared to wild-type controls due to prevention of oxidative stress and improved endothelial function [70,71].

### 7.5 Cell cycle regulators, senescence and autophagy

In culture, vascular cells respond to prolonged series of replication and stresses by eventually entering an irreversible growth arrest or senescent state [72]. After the Hayflick limit, cells enter an irreversible cell cycle arrest in the G1 phase of the cell cycle and no longer respond to growth stimuli. This phenomenon is called replicative senescence and occurs in vascular ageing [73]. Senescent cells have a distinct phenotype—they are large and flattened, express specific markers (β-galactosidase), overexpress cell cycle molecular markers (p16 and p21), form heterochromatic foci (yH2AX) and accumulate lipofuscin, a non-degradable fluorescent compound [74]. Whilst the molecular mechanisms underlying cellular senescence have been the focus of numerous studies, the impact of senescence in vivo has yet to be fully established, especially since some studies show increased rates of vascular cell proliferation in ageing and longevity [75,76].

Considering the remarkable plasticity of vascular smooth muscle cells, there is a requirement for tight control of transcriptional, metabolic and ultrastructural processes, events that are coordinated through autophagy. Autophagy is the basic cellular mechanism that involves cell degradation of unnecessary or dysfunctional molecules through lysosomes [77]. In the vasculature, changes in autophagy have been observed in experimental ageing [78].

### 7.6 Mitogen-activated protein kinases (MAPK)

Protein kinases are major regulators of signal transduction that catalyse the phosphorylation of other proteins, thus regulating their activity. Primary targets of protein kinases include transcription factors which modulate intracellular signalling via specific alteration of downstream gene expression/activity [79]. A key group of protein kinases in the vasculature are the serine/threonine sub-family, which act by promoting phosphorylation of the OH group of serine or threonine residues on target proteins [80]. Mitogen activated protein kinases (MAPKs) represent a large family of proteins important in signal transduction within the cardiovascular system, where they are involved in regulation of a number of biological processes, such as cell migration, survival, apoptosis, proliferation, contraction and differentiation. MAPK signalling is promoted by many stimuli including GPCR activation, receptor tyrosine kinases, oxidative stress and growth factors, and comprises a number of sequentially acting kinases which ultimately result in phosphorylation and activation of terminal effector kinases, thereby transducing specific cellular actions [80,81]. Several MAPK family subgroups have been identified, of which the major mammalian types appear to be ERK1/2, c-Jun NH2-terminal kinases (JNK1, 2 and 3) and p38MAPK (α, β, δ and γ), which play key roles during cardiovascular development and vascular function [82,83]. Several studies have demonstrated an age-dependent increase in MAPK activation in vascular tissue [84,85].

### 7.7 Oxidative stress in vascular ageing

**Fig. 3:**
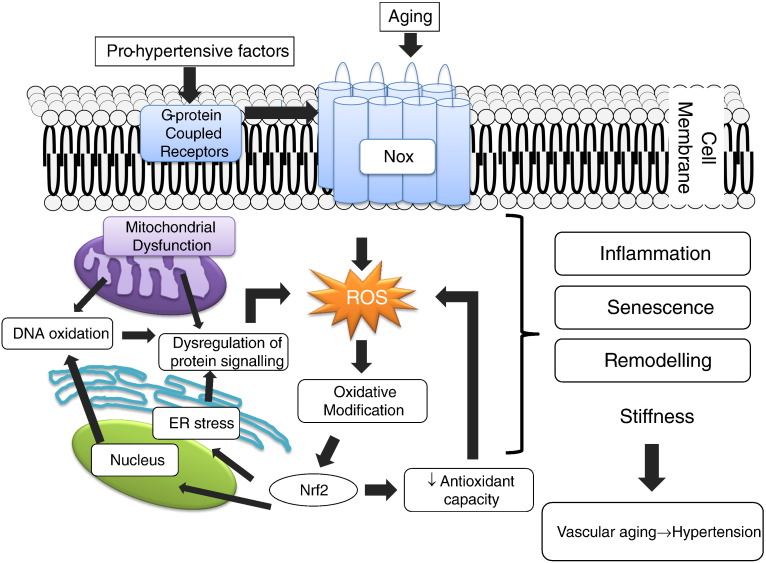
Role of reactive oxygen species (ROS) in vascular processes associated with ageing and hypertension. Pro-hypertensive factors, such as angiotensin II and endothelin-1, and biological ageing, increase ROS production in vascular cells. An increase in the levels of ROS lead to oxidation of proteins and DNA, affecting cell signalling and inducing injurious responses, such as inflammation, senescence, fibrosis, calcification, and hypertrophy in the vasculature. Oxidation of transcription factors that regulate the anti-oxidant capacity in vascular cells, such as Nrf2, are also affected by oxidation leading to decreased activity. Sources responsible for the increase in ROS generation and oxidative modification of cellular molecules are the mitochondria, NADPH oxidases (Nox) and endoplasmic reticulum (ER) stress.

Common to many of the molecular and cellular processes described above that underlie changes in the vasculature with ageing is oxidative stress [86]. The concept that ROS are linked to ageing was suggested in 1956 by Harman when he proposed the Free Radical Theory of Ageing, stating that the accumulation of free radicals during ageing causes the damage of biomolecules by these ROS and the development of pathological disorders promoting cell senescence and organism ageing [87,88]. Such processes are evident in vessels associated with ageing and with hypertension [87,88]. Excessive production of ROS and reactive nitrogen species (RNS) leads to oxidative modification of proteins, DNA and lipids, which accumulate in cells leading to impaired cellular and vascular function. In addition increased vascular ROS levels, together with decreased eNOS-generated NO, compromise the vasodilatory actions of NO and promote the formation of injurious peroxynitrite, processes observed in aorta of aged rodents [89]. Oxidative stress is critically involved in many of the molecular events of vascular ageing, including: (1) increased pro-inflammatory responses in vascular cells, (2) vascular dysfunction through oxidative modification of structural and functional proteins regulating vascular contraction/relaxation, fibrosis and calcification, (3) altered calcium homeostasis in vascular cells, 4) activation of redox-sensitive pro-inflammatory and pro-fibrotic transcription factors, and (4) activation of molecular mechanisms leading to senescence and autophagy in endothelial and vascular smooth muscle cells (Fig. 3). The fact that SOD mimetics, such as tempol, normalise endothelial dysfunction in old rodents supports a role for increased superoxide anion levels in age-related endothelial impairment [90].

Changes in cellular anti-oxidant systems are also important. The expression and activity of antioxidant enzymes, including SOD, decline as tissues age. Decreased anti-oxidant capacity is further promoted by downregulation of Nrf2, the master transcription factor regulating anti-oxidant genes [91]. These processes are accompanied by chronic low-grade inflammation mediated by redox-sensitive NFκB, which is upregulated in aged vessels [92].

Multiple oxidases generate ROS in the vascular wall and endothelium, including NADPH oxidases (Nox), xanthine oxidase, uncoupled NOS and mitochondrial oxidases. Of these, mitochondria seem to play a major role in processes related to ageing. Noxs, of which there are 7 isoforms (Nox1-5, Duox1, Duox2), have also been shown to contribute to oxidative stress in vascular ageing [93–95]. In particular, in aged spontaneously hypertensive rat aortas, expression of Nox1 and Nox2, but not of Nox4, was increased. This Nox upregulation was associated with endothelial dysfunction, which was reversed by VAS2870, a Nox inhibitor [96]. Noxs appear to be more important in pathological vascular remodelling associated with hypertension and cardiovascular diseases [97–99]. Vascular xanthine oxidase and cytochrome P450 epoxygenases seem to be less important, since expression and activity of these systems is not altered with ageing in humans [100].

With biological ageing, mitochondria become dysfunctional leading to reduced energy production and increased ROS formation. Mechanisms related to mitochondrial dysfunction during ageing include decreased ATP synthesis, increased apoptosis and mutations of mitochondrial DNA by oxidation [101]. During ageing, the electron flow in mitochondria decreases, altering the oxygen consumption and inducing ROS generation [101]. The pro-oxidative environment increases mitochondrial DNA damage, leading to further dysfunction of the respiratory chain and more ROS production. Consequently, the rate of apoptosis increases, releasing an excessive amount of ROS into the cytosol, further contributing to oxidative stress and vascular cell damage.

### 7.8 Endoplasmic reticulum stress in vascular ageing

Prolonged perturbation of the endoplasmic reticulum (ER) leads to ER stress and unfolded protein response (UPR) and contributes to pathogenic processes associated with vascular damage and endothelial dysfunction [102]. The ER is an important site where proteins are folded and post-translation modifications occur. It is also a site for Ca^2 +^ storage and cholesterol/lipid biosynthesis. Due to the large amount of unfolded protein in the ER, a control system that avoids protein aggregation and accumulation of unfolded proteins is necessary. In experimental models of ageing, the expression and activity of ER chaperones or folding enzymes decay, whilst oxidative damage, such as carbonylation, is exacerbated, leading to accumulation of misfolded/unfolded proteins and ER stress. This activates signalling mechanisms that are part of the UPR. Induction of ER stress leads to endothelial cell apoptosis, but not senescence, implicated in endothelial dysfunction in ageing [103,104]. Inhibition of ER stress has been suggested as a novel therapeutic strategy to ameliorate vascular dysfunction during ageing [105]. However such approaches still require further investigation.

### 7.9 Vascular changes in hypertension recapitulate those in ageing

Many of the signalling pathways associated with vascular changes during ageing are also activated in hypertension leading to endothelial dysfunction, vascular inflammation, remodelling and increased arterial stiffness. With normal physiological ageing the process is gradual and regulated but in itself represents a strong and independent risk factor for hypertension and future cardiovascular events [106]. In susceptible individuals, due to genetic, environmental or in-utero factors 9fetal programming), processes underlying vascular changes are accelerated leading to ‘early vascular ageing’, which predisposes to cardiovascular disease. Numerous risk factors amplify the process of arterial ageing, including atherosclerosis, smoking, increased sodium intake and hypertension, due, in part, to increased oxidative stress, activation of pro-inflammatory and pro-fibrotic signalling pathways and upregulation of the renin–angiotensin–aldosterone system. As with ageing, experimental and human hypertension show a reduction in endothelium-dependent vasodilation, decreased NO bioavailability, NO synthase uncoupling, increased oxidative stress, telomere shortening and associated endothelial dysfunction. In arteries from aged humans, non-human primates and rodents, expression of the AT_1_R is increased and sensitivity of the mineralocorticoid receptor to aldosterone is enhanced, phenomena that are also observed in hypertension [107–109]. Ang II promotes vascular calcification, inflammation, cell proliferation and fibrosis and mimics age-associated vascular remodelling in young rodents [110–112]. In large arteries these molecular and cellular processes manifest as increased arterial stiffness, which is a major contributor to elevated central blood pressure leading to isolated systolic hypertension, common in the elderly. Exactly what triggers these cellular and vascular events remains unclear, and it is difficult to dissect out the ‘ageing effect’ from the ‘blood pressure effect’. This ‘conundrum of arterial stiffness, elevated blood pressure and ageing’ has recently been reviewed by AlGhatrif and Lakatta [113], who concluded that vascular properties depend on the net effect of multiple factors that are interdependent and which change with ageing over a lifetime.

### 7.10 Effects of pro-hypertensive stimuli on vascular ageing: the renin–angiotensin–aldosterone system (RAAS)

The RAAS plays an important role in functional, structural and mechanical changes of the vasculature that occur with ageing and hypertension [114]. This occurs through increased signalling via the AT_1_ receptor. Expression of various components of the RAS, including angiotensinogen, chymase, angiotensin converting enzyme (ACE) and the AT_1_ receptor is increased in arteries of aged rodents and humans [8,9]. To further support a role for the RAAS in the ageing process and during hypertension are studies showing that ACE inhibitors and AT_1_ receptor blockers decrease ageing-associated vascular damage. Mice treated with enalapril or losartan demonstrated vasoprotection and an increase in life span [115,116]. Processes associated with these effects involve upregulation of NOS activity and increased NO production. An increase in antioxidant defences, such as SOD and glutathione, is another mechanism involved in the anti-ageing effects of the inhibition of the RAAS system, leading to an increase in NO bioavailability [117]. Moreover, lifelong treatment of young stroke-prone spontaneously hypertensive rats, with AT_1_ receptor blockers doubles the lifespan by improving endothelial function and alleviating complications of hypertension [118]. To further support a role for Ang II/AT_1_ receptor in oxidative stress, vascular injury and ageing, studies in mice with targeted disruption of the Agtr1a gene, which encodes the AT_1_A receptor, resulted in prolonged life span [119]. Agtr1a −/− mice developed less cardiac and vascular injury and oxidative damage was reduced compared with wild-type counterparts. The longevity phenotype was associated with an increased number of mitochondria and upregulation of pro-survival genes.

Clinical and experimental studies demonstrate that many pro-hypertensive systems influence processes of vascular ageing, including aldosterone, ET-1 and growth factors [120–124]. Arteries from aged rodents demonstrate upregulation of these systems, leading to stimulation of signalling pathways, oxidative stress, and activation of pro-inflammatory transcription factors, which promote a shift of endothelial and vascular smooth muscle cells to an ageing phenotype. On the other hand, infusion of Ang II, aldosterone or ET-1in young animals, recapitulates arterial changes observed in aged animals.

### 7.11 Effects of novel anti-hypertensive factors on vascular ageing

NO is a potent vasodilator produced by endothelial cells that mediates vascular relaxation and thus plays a critical role in the regulation of blood pressure. Abnormalities in endothelial production of NO occur in hypertension and are due, in large part, to decreased eNOS activity [125]. NO donors such as glyceryl trinitrate (GTN) have been shown to possess anti-hypertensive properties [126] and evidence is emerging that NO and NO donors could confer beneficial effects on the phenotypic alterations that occur in the vasculature with ageing. For example, NO prevents differentiation of VSMCs into osteoblastic cells by inhibiting TGF-β [127]. The NO donor *S*-nitroso-penicillamine significantly reduces endothelial cell senescence and age-dependent inhibition of telomerase activity [128,129].

The gaseous messenger hydrogen sulphide (H_2_S) produced by cystathionine g-lyase (CSE) or cystathionine b-synthase (CBS) has recently emerged as a novel antihypertensive factor based on the observations that exogenous H_2_S is vasoprotective in pulmonary hypertension [130] and that it reduces systemic blood pressure by improving endothelial function [131]. CSE-deficient mice, have increased blood pressure and impaired endothelial function [132]. Mouse embryonic fibroblasts from CSE knockout mice display accelerated cellular senescence and increased expression of p53 and p21, processes which were prevented by NaHS treatment. NaHS also enhanced Nrf2 nuclear translocation, and stimulated mRNA expression of Nrf2-targeted downstream anti-oxidant genes in this system, highlighting an important interplay between cellular ageing, senescence and oxidative stress [133]. Attenuation of endothelial cell senescence by H2S occurs through modulation of SIRT1 activity [134].

Plasma levels of H_2_S in humans decline with age [135] and several studies have shown that H_2_S protects against free radical-induced damage and exerts beneficial effects on age-associated diseases [136]. Several lines of evidence indicate that these beneficial effects may extend to vascular ageing, characterised by positive effects on many of the phenotypic vascular alterations that occur with advancing age. For example, the production of H_2_S is decreased in a rodent model of vascular calcification with the addition of H_2_S ameliorating this phenotype [137]. Further, in vascular smooth muscle cells, H_2_S was found to inhibit calcium deposition in the extracellular matrix and suppress induction of osteoblastic transformation genes [138].

In endothelial cells stimulated with TNF-α, NaHS (H2S donor) suppressed pro-inflammatory responses by reducing the TNF-induced increase in expression of intercellular adhesion molecule-1 (ICAM-1), vascular cell adhesion molecule-1 (VCAM-1), P-selectin and E-selectin. Furthermore, TNF-α-induced NF-κB was decreased in the presence of NaHS [139]. H_2_S donors (NaHS and Na_2_S) can inhibit leukocyte adherence in mesenteric venules whilst inhibition of endogenous H_2_S synthesis promotes leukocyte adhesion and vascular inflammation [140].

### 7.12 Vascular damage in hypertension may be independent of ageing

Although there are many signalling pathways and functional and structural characteristics that are common in vessels during ageing and hypertension, these processes are dynamic and change throughout life and as such may not necessarily be superimposable. For example, with advancing age arterial stiffness and blood pressure start to diverge rather than parallel [141]. Also, circulating markers of inflammation including sVCAM-1, IL-6 and MCP-1 increase with age but do not necessarily correlate with elevations in blood pressure [142]. Furthermore, with ageing, aortic calcification is independently predictive of subsequent vascular morbidity and mortality beyond established risk factors with no evident correlation between calcification and systolic BP [143].

There is also some evidence to show that structural alterations in the vascular wall occur before the development of hypertension. For example rates of pulse wave velocity (PWV) increase are accelerated with both advancing age and elevated blood pressure. However, the effect of blood pressure on PWV increase occurs primarily during the prehypertensive phase and suggests that these vascular alterations precede the phase of established hypertension [8,144].

### 7.13 Lessons learned from children with hypertension

Vascular changes that occur with ageing may be independent of biological ageing in hypertension. This is highlighted in studies that have examined vascular function and arterial structure in children with hypertension. Endothelial dysfunction, arterial stiffening and structural alterations of the arterial wall may precede evidence of high blood pressure, as quantified by systolic and diastolic blood pressure, and may be independent of the ageing process [145]. This is evidenced by the findings that vascular injury is already present in children with mild hypertension, processes that are exaggerated as hypertension becomes more severe [146,147]. Functional alterations, including reduced endothelium-dependent vasorelaxation and decreased elasticity, seem to precede vascular structural changes. In obese pre-pubertal children, impaired brachial endothelial and vascular smooth muscle function is present without concomitant increase in carotid intima-to-media thickness. Functionally these changes lead to decreased vascular distensibility and increased rigidity or stiffness. Arterial stiffness, as assessed by measurement of PWV, is increased in children with type 1 diabetes and in children with hypertension. Increased arterial stiffness in childhood hypertension is an important risk factor for severe hypertension and cardiovascular complications later in life. Results from the Amsterdam Growth and Health Longitudinal Study indicate that individuals with stiffer carotid arteries at 36 years of age were characterised during adolescence by increased blood pressure and increased PWV [148]. Factors that have been implicated in vascular dysfunction in childhood hypertension include activation of the sympathetic nervous system, adipokines, upregulation of the RAAS and increased oxidative stress, processes that also underlie physiological vascular ageing and EVA in adult hypertension [149–152].

Taken together, emerging experimental and clinical evidence indicates that although the molecular and cellular processes that characterise the vascular phenotype in hypertension resemble those that occur with normal healthy ageing, age per se may not be a critical factor. However, ageing may be a compounding factor that amplifies vascular injury that occurs with blood pressure elevation. In the presence of other co-morbidities, such as diabetes, dyslipidemia, smoking and obesity, these processes may be further exaggerated.

## 8 Summary and conclusions

Ageing is associated with a progressive deterioration in endothelial function, vascular remodelling, inflammation and increased arterial stiffness. Processes underlying these processes include activation of pro-inflammatory transcription factors, oxidative stress, cell senescence and apoptosis, aberrant signalling cascades and a shift from a vasoconstrictor to a proliferative vascular cell phenotype. Many of these phenomena are also relevant in the pathophysiology of hypertension, which is characterised by a vascular phenotype of impaired endothelium-dependent vasorelaxation, arterial remodelling, increased stiffness and vascular inflammation. Through such vascular changes, ageing and hypertension are closely interlinked: ageing promotes hypertension and pro-hypertensive factors promote vascular ageing. Whilst many of the molecular processes and signalling pathways contributing to vascular dysfunction are common in ageing and in hypertension, biological age per se, may not be a fundamental factor, since vascular damage is already present in children and young adults with hypertension. A better understanding of the vascular biology of ageing will facilitate development of strategies to promote healthy vessels and suppress age-associated changes, especially in pathological conditions. Such approaches could prevent or ameliorate vascular damage in hypertension and hence reduce cardiovascular diseases, commonly linked to ageing.

## Disclosures

There are no disclosures to declare.
